# Gordon’s Functional Health Patterns and Their Association with Patient and Organizational Outcomes: A Scoping Review

**DOI:** 10.3390/healthcare14091144

**Published:** 2026-04-24

**Authors:** Clarissa Santos de Lima Araújo, Larissa Maiara da Silva Alves Souza, Agueda Mª Ruiz Zimmer Cavalcante, Janaína Guimarães Valadares, Flaviana Vely Mendonça Vieira, Dorothy Jones, Natália Del Angelo Aredes, Luca Bertocchi

**Affiliations:** 1 Nursing College, Federal University of Goiás, Rua 227, Viela Q. 68, S/N—Setor Leste Universitário, Goiânia 74605-080, GO, Brazil; clarissa.araujo@discente.ufg.br (C.S.d.L.A.); janainavaladares@ufg.br (J.G.V.); flavianavieira@ufg.br (F.V.M.V.); naredes@ufg.br (N.D.A.A.); 2 Nursing College, Federal University of Sao Paulo, Rua Napoleão de Barros, 754-2° Andar–Vila Clementino, São Paulo 04024-002, SP, Brazil; larissa.alves91@live.com; 3 The Marjory Gordon Program for Clinical Reasoning and Knowledge Development, William F. Connell School of Nursing, Boston College, Chestnut Hill, MA 02467, USAluca.bertocchi@asugi.sanita.fvg.it (L.B.); 4 Hematology Unit, Department of Oncology, Trieste University Hospital (ASUGI), 34129 Trieste, Italy

**Keywords:** Gordon’s functional health patterns, nursing assessment, nursing process, standardized nursing terminologies, electronic health records, clinical decision support systems, NANDA-I, outcomes, scoping review

## Abstract

**Background/Objectives**: Nursing assessment frameworks play a critical role in guiding holistic patient evaluations, standardizing documentation, and supporting organizational quality and safety initiatives. Among these, Gordon’s Functional Health Patterns (FHPs) offer a comprehensive and widely used framework for nursing assessment. However, no review has synthesized evidence on their association with outcomes. This scoping review aimed to map evidence on the use of FHPs in relation to patient and organizational outcomes, and to examine their integration into electronic health records (EHRs) and the analytical methods employed. **Method**: A scoping review was conducted following Joanna Briggs Institute methodology and PRISMA-ScR guidelines. PubMed, CINAHL, and Scopus were searched for quantitative primary studies reporting associations between FHPs and outcomes, and the final search was conducted on 22 March 2024. Three reviewers independently screened abstracts and full texts and extracted data. **Results**: Seven studies met the inclusion criteria. FHPs’ use was associated with improvements in several patient outcomes, including quality of life, psychological well-being, clinical parameters, self-management, dependency level, and functional performance. Organizational outcomes included reduced hospital readmission rates and a positive association between FHP-derived nursing diagnoses and nursing workload. Most studies used standardized nursing terminologies such as NANDA-I, NOC, or NIC, in conjunction with FHPs. Over half of the studies used EHR-based nursing documentation, reflecting increasing digital integration and enabling more structured and interoperable nursing data. Methodological approaches varied widely: most studies used associative analyses, two employed experimental designs, and one investigated the predictive utility of FHP-based assessment data. **Conclusions**: FHPs provide a structured framework for nursing practice with potential benefits for patient and organizational outcomes. Their increasing integration into EHRs supports standardized documentation and data-driven nursing practice, enhancing assessment quality, diagnostic accuracy, and the availability of structured data for clinical and managerial decision-making in health information systems. Further experimental and longitudinal research is needed to strengthen causal evidence and guide implementation.

## 1. Introduction

The nursing process is a systematic method of critical thinking, problem-solving, and scientific reasoning that guides professional nursing care [1]. The use of standardized nursing terminologies (SNTs) enhances the quality and safety of care by promoting consistency in communication, documentation, and clinical decision-making [2,3]. Among the various SNTs currently in use, the most widely adopted are NANDA-International (NANDA-I), the Nursing Outcomes Classification (NOC), and the Nursing Interventions Classification (NIC) [2,4]. These terminologies are formally recognized by the American Nurses Association and primarily support the diagnostic, intervention, and outcome phases of the nursing process.

Nursing assessment represents the first and foundational phase of the nursing process and consists of the systematic, continuous collection, analysis, and interpretation of data related to an individual’s health status [5]. A comprehensive and accurate nursing assessment reflects patients’ functional status and health needs, providing the basis for appropriate nursing diagnoses, interventions, and outcome evaluation [6]. In contrast, incomplete or inconsistent assessment data may compromise subsequent phases of the nursing process and negatively affect care quality and patient safety. Moreover, nursing assessment plays a critical role in identifying risks for adverse outcomes and supporting preventive and proactive care [7]. When systematically structured, nursing assessment data can also support clinical nursing information systems, enable secondary use of data, and contribute to organizational performance monitoring and quality improvement.

Despite the widespread adoption of SNTs, a standardized, universally accepted framework for the nursing assessment phase remains lacking [8]. While SNTs effectively structure diagnoses, outcomes, and interventions, the assessment phase often relies on heterogeneous tools and instruments, limiting comparability, data reuse, and the visibility of nursing contributions [9,10]. Numerous studies have proposed or evaluated standardized nursing assessment tools within different conceptual frameworks [11,12,13]; however, the lack of consensus on assessment frameworks continues to hinder the integration of nursing assessment data into electronic health records (EHRs) and broader health information infrastructures.

Within this context, Gordon’s Functional Health Patterns (FHPs) offer one of the most comprehensive and widely used frameworks for organizing nursing assessment data in both clinical practice and information systems [8]. Developed by Marjory Gordon in 1994, the model comprises 11 functional health patterns (*Health Perception-Health Maintenance*, *Nutritional–Metabolic*, *Elimination*, *Activity-Exercise*, *Sleep-Rest*, *Coping–Stress*, *Self-Concept –Self-Perception*, *Cognitive-Perceptual*, *Role–Relationship*, *Sexuality–Reproductive*, and *Value–Belief patterns*) that enable holistic evaluation of individuals, families, and communities [14,15]. Each pattern organizes assessment data related to functional abilities, risks, and patient strengths, supporting a systematic and comprehensive approach to nursing assessment [14,15]. By organizing assessment data across interconnected health domains, the FHP framework supports clinical reasoning and facilitates identification of patient strengths, vulnerabilities, and care priorities [16]. Importantly, Gordon’s FHPs have been used frequently in conjunction with NANDA-I nursing diagnoses, thereby enhancing diagnostic accuracy by systematically identifying defining characteristics, signs, and symptoms [8].

Several other structured nursing assessment frameworks, such as *Henderson’s 14 Fundamental Needs* and the *Roper-Logan-Tierney Model of Living*, also provide systematic approaches for organizing patient assessment [8,9]. Gordon’s FHPs are distinguished by their holistic focus on functional health domains and their strong alignment with SNTs and EHRs structures, making them particularly relevant for contemporary clinical documentation and data-driven nursing practice [8,9].

Although Gordon’s FHPs have been used in previous studies [16,17], no comprehensive synthesis of their association with patient and organizational outcomes exists [8]. Therefore, this scoping review aimed to address this gap by mapping the evidence on the association of using Gordon’s FHPs in these areas. Additionally, the review examines the integration of Gordon’s FHPs into EHRs and the main analytical methods used in these studies.

## 2. Materials and Methods

This scoping review was conducted using the methodological framework proposed by the Joanna Briggs Institute (JBI) guidelines [18] and is reported in accordance with the PRISMA extension for Scoping Reviews (PRISMA-ScR) guidelines [19] (Supplementary Table S1). The protocol was prospectively registered on the Open Science Framework (OSF) platform (https://osf.io/3vkzw/, accessed on 28 February 2024) to ensure transparency and methodological rigor.

### 2.1. Review Question

The review question was developed using the Population, Concept, and Context (PCC) framework, recommended by JBI to guide scoping review eligibility criteria (Table 1). The primary research question was “What evidence exists regarding the use of Gordon’s FHPs in nursing practice, and what patient and organizational outcomes are associated with their use?”

Studies were eligible if they employed Gordon’s FHPs for assessment in any healthcare setting, population, or health condition. Eligible full-text articles were published in English, Portuguese, or Italian. We included quantitative primary research examining associations between FHP-based assessment and patient or organizational outcomes. We excluded qualitative studies, case studies, case reports, discussion papers, reviews and studies without outcome measures. Qualitative designs were not included because they do not provide quantifiable outcome data relevant to this outcomes-focused review. No time limit was applied.

### 2.2. Search Strategy

A preliminary exploratory PubMed search was conducted to identify relevant English-language terms. The same search was then conducted in CINAHL and Scopus. A final search strategy across the three databases was conducted on 22 March 2024. The research was organized around two concepts derived from the research question: nursing assessment (in this case, Gordon’s FHPs) and outcomes, connected with the Boolean operators AND or OR. Based on these concepts, key terms were identified and used to develop database-specific search strategies (Supplementary Table S2).

### 2.3. Study Selection and Data Extraction

After completing the database searches, references were imported into Rayyan^®^ (https://www.rayyan.ai/) for citation management, and duplicates were removed. Two independent reviewers then screened titles and abstracts against the predefined inclusion criteria. Full texts of potentially eligible articles were subsequently retrieved and assessed for eligibility. When full texts were unavailable through institutional access, authors or publishers were contacted; studies for which the full text could not be obtained were excluded at this stage.

A structured data extraction tool, developed in alignment with JBI guidance, was applied to all studies meeting the inclusion criteria. Data extraction included: author, year, country, study design, setting, sample characteristics, type of health record used, SNTs, modality of FHP processing, patient and organizational outcome measurements, follow-up time, key results, and the main type of analysis. Any disagreements in the study selection process were resolved by a third reviewer. Data extraction was carried out by three researchers, who harmonized and condensed the information following JBI’s recommended data-charting procedure.

## 3. Results

A total of 1033 records were retrieved from the three electronic databases. After duplicate removal, 1007 abstracts were screened for eligibility. Twenty-six articles were assessed for inclusion.

**Figure 1:**
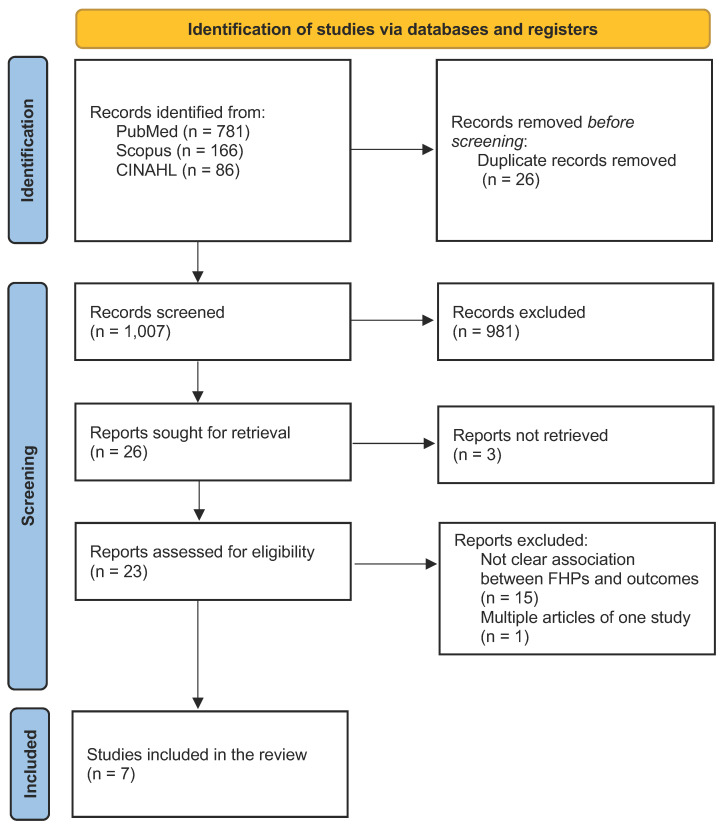
Preferred Reporting Items for Systematic Reviews and Meta-Analyses 2020 (PRISMA) flow diagram of included studies. CINAHL, Cumulative Index to Nursing and Allied Health Literature; FHPs: functional health patterns; n, number.

During full-text review, 19 studies were excluded due to: (a) lack of a clear association between Gordon’s FHPs and outcomes (n = 15), (b) full text not accessible (n = 3), or (c) multiple reports of the same study (n = 1). Finally, a total of seven studies were included in the scoping review (Figure 1). The selected publications are summarized in the data charting table (Table 2).

### 3.1. Characteristics of Included Studies

The studies were published between 1997 and 2023, with most published in the last decade. Most were conducted in Spain (42.8%), Turkey (28.5%), Mexico (14.2%), and Canada (14.2%). Most studies recruited patients through convenience sampling (71.4%) or random sampling (28.5%). Over half of the studies (57.1%) were observational, whereas 42.9% were experimental or quasi-experimental. The main settings were hospitals (n = 4; 57.1%) and primary healthcare centers (n = 3; 42.9%). More than 50,000 adult participants were recruited (range: 68–51,374). Most studies (71.4%) used SNTs associated with Gordon’s FHPs: NANDA-I, NOC, and NIC. Table 2 presents the key characteristics of the included studies.

### 3.2. Outcomes and Associations of Gordon’s FHPs

Data from the seven articles were synthesized into narrative and tabular formats. Table 2 summarizes the narrative results. Most studies (n = 5) examined only patient outcomes [20,21,23,24,25], one explored an organizational outcome [26], and one examined both patient and organizational outcomes [21]. In the longitudinal studies, follow-up time ranged from 30 days to two years.

### 3.3. Gordon’s FHPs and Patient Outcomes

Patient outcomes were organized into seven categories (Table 3). The most frequently studied outcomes were clinical (n = 2), self-management/compliance (n = 2), and dependency level (n = 2); the other investigated outcomes were mortality (n = 1), quality of life (n = 1), psychological outcomes (n = 1), and functional performance (n = 1) (Table 3).

**Quality of life and mortality**: Türen & Enç [21] reported that, in a randomized sample of 120 adult patients with heart failure, using Gordon’s FHPs booklet was associated with a significantly higher quality of life at 30 days (*p* = 0.001) and a lower mortality rate (although this difference did not reach statistical significance).

**Psychological outcomes**: Temel & Kutlu [22], in a convenience sample of 68 adults with depression, found that the interventional group using Gordon’s FHPs experienced a significant reduction in depressive symptoms and hopelessness and an increase in self-efficacy.

**Clinical outcomes**: Cardenas-Valladolid et al. [23], in a two-year prospective study of 23,488 patients with diabetes, found that using Gordon’s FHPs, NANDA-I, and NIC improved blood glucose, blood pressure, and lipid control. In a randomized controlled study of 171 neurological patients, fewer urinary problems were observed in the intervention group, which received an educational intervention (personalized nurse counseling) based on Gordon’s FHPs [20].

**Self-management/compliance**: Cazares Miranda [20] used an educational intervention based on Gordon’s FHPs and reported a significantly higher treatment adherence score than the control group. Lizcano-Álvarez [24], in a convenience sample of 132 adult patients with acute coronary syndrome, reported a significant increase in all NOC indicators (self-management, compliance behavior) from pre- to post-intervention, using 11 nursing consultation programs based on Gordon’s FHPs.

**Dependency Level and Functional Performance**: Positive associations between Gordon’s FHP model and dependency level were reported by Cazares Miranda [20] and Brito-Brito [25]. Brito-Brito [25], in a convenience sample of 51,374 highly complex chronic adult patients, in which Gordon’s FHPs, NANDA-I, NOC and NIC were used, showed a strong significant difference (*p* < 0.001) between dependency levels (measured with a dependency level classification) and physical activity/exercise, while there was a moderate significant difference (*p* < 0.001) between dependency levels and health perception/health management, elimination, and cognition/perception. Cazares Miranda [20], using an educational intervention based on Gordon’s FHPs in neurological patients, showed that the Barthel index had a significantly higher score of independence (effect size, 23%; *p* < 0.05). Regarding functional performance, Cazares Miranda [20] reported a favorable odds ratio in recreational and productive activities compared to the control group.

### 3.4. Gordon’s FHPs and Organizational Outcomes: Readmission and Amount of Nursing Care

The organizational outcomes included readmission and the number of nursing care indicators (Table 3). Türen & Enç [21], using Gordon’s FHPs, reported a reduction in 30-day hospital readmission rates among heart failure patients. O’Brien-Pallas [26] found a positive linear relationship between the number of nursing diagnoses selected within each Gordon’s FHP (except coping-stress tolerance) and the average workload (an indicator of the amount of nursing care). Finally, Türen & Enç [21] reported that the emergency room visit rate was higher in the control group that did not use Gordon’s FHPs, although the difference was not statistically significant (*p* = 0.12).

### 3.5. Integration of Nursing Assessment into Electronic Health Records

Approximately 57% of the included studies (n = 4) reported the use of EHRs to document nursing assessment data based on Gordon’s FHPs. Three of these four studies were conducted within the last decade [21,24,25]. In the three remaining studies, the type of nursing documentation used was not specified.

### 3.6. Methodological Characteristics of Studies

Most studies examined associations between Gordon’s FHPs and patient or organizational outcomes. Fewer studies investigated causal relationships (n = 2), both using experimental designs, and only one study explored the predictive utility of FHPs. Regarding analytical methods, four studies primarily employed multivariate analyses (e.g., logistic regression, ANOVA, ANCOVA, Cox regression), while three applied bivariate analyses in combination with descriptive statistics. Across studies, FHPs were generally treated as independent variables, with patient or organizational outcomes specified as dependent variables.

## 4. Discussion

The findings of this scoping review provide a comprehensive synthesis of the available literature on the use of Gordon’s FHPs across different healthcare contexts, with a focus on patient and organizational outcomes. Across the included studies, the use of Gordon’s FHPs was most frequently associated with favorable patient outcomes, including quality of life, as well as organizational outcomes, such as readmissions. In addition, more than half of the studies used EHRs, indicating an increasing integration of FHP-based assessment into digital documentation systems and highlighting its potential to support structured nursing data and inform evidence-based practice. To the best of our knowledge, this is the first scoping review to systematically map and synthesize the existing evidence on Gordon’s FHPs on healthcare outcomes.

### 4.1. Patient Outcomes

Studies using Gordon’s FHPs may contribute to improving patient outcomes, including clinical outcomes, self-management, dependency level, mortality, quality of life, and psychological and functional performance.

Two studies [20,23] found that using Gordon’s FHPs yielded significant improvements in clinical outcomes. In a randomized study with neurological patients, Gordon’s FHP served as a guide for personalized nurse counseling. Patients without problems with the functional health pattern of elimination were 24 times less likely to develop a urinary tract infection than those in the control group, which is common, especially in patients with a spine injury [20]. This result highlights the advantages of using Gordon’s FHPs in nursing assessment to identify risks and to support the subsequent implementation of a personalized care plan based on specific interventions for individual patients.

As for self-management outcomes, Lizcano-Álvarez [24] conducted research in primary care settings and reported that, for post-myocardial infarction patients and their families, role performance and relationship patterns can be more easily assessed, allowing for the identification of related issues and, consequently, early interventions. These interventions are only possible through problem identification, which relies on nurses’ perspectives and the theoretical framework applied during the assessment and history-taking process [24].

Dependency level was studied by Brito-Brito [25] in an observational study of complex chronic patients in primary care settings. It used a semi-structured assessment based on Gordon’s FHPs, NANDA-I, NOC, and NICs. The results found an association between dysfunction across various health patterns and dependency status, which may help improve care and quality of life for these people. These findings are consistent with previous reviews on the impact of SNTs on outcomes, although our scoping review focused only on assessment [2,7,27]. However, these results should be interpreted with caution, given the substantial heterogeneity of the interventions and outcomes used. Each study employed distinct interventions and assessed different outcomes, which precluded quantitative synthesis or meta-analysis.

Türen & Enç [21] demonstrated that the experimental group applying Gordon’s FHPs had a significantly better quality of life (Minnesota Living with Heart Failure Questionnaire) and a lower 30-day event-free survival rate (Kaplan–Meier curve), indicating fewer adverse health events among those receiving FHP-based care. A study of 12-month mortality risk among older adults reported similar findings [28]. These results suggest that the use of Gordon’s FHPs challenges the limitations of a biomedical assessment model, which views patients primarily through disease mechanisms and their clinical manifestations. By organizing assessment data around functional and psychosocial patterns, FHPs enable nurses to detect clinically relevant issues that the biomedical assessment often misses. Evidence shows that nursing diagnoses derived from such holistic assessment—such as *imbalanced nutrition: less than body requirements* and *death anxiety*—serve as independent predictors of mortality, even after controlling for established prognostic indexes [29]. The application of functional health patterns, therefore, enhances the visibility and prognostic value of nursing assessment and broadens the scope of nursing care.

Temel & Kutlu [22] found that in individuals with depression, using Gordon’s FHP in the intervention group resulted in a significant reduction in depressive symptoms and hopelessness while increasing patients’ self-efficacy in coping with depression. The model’s systematic approach not only reduces health problems but also ensures more efficient and organized care.

### 4.2. Organizational Outcomes

Studies using Gordon’s FHPs have suggested that this structured approach to patient assessment can reduce readmission rates, as evidenced in heart failure research [21]. While the FHPs assessment itself may not be the primary driver of reducing readmissions, integrating it with tailored nursing diagnoses could be an effective way to improve patient outcomes. This approach aligns with the notion that comprehensive assessments can reveal key patient needs, which, when addressed through specific nursing interventions, may reduce the likelihood of rehospitalization [2].

An essential factor in successfully implementing these frameworks is understanding and managing nursing staff work demands. High work demands are known to have significant effects on both nurse health and patient care outcomes, emphasizing the need for supportive organizational structures [30]. Monitoring the intensity and quality of nursing care provided is thus critical. For instance, O’Brien-Pallas et al. [26] found a positive linear relationship between the number of nursing diagnoses within each FHP (except for coping-stress tolerance) and the average workload. This highlights that a higher number of targeted nursing diagnoses correlates with increased workload, which may imply more comprehensive patient care but also stresses the need for adequate staffing and support to maintain quality care.

Furthermore, studies suggest that nursing diagnoses documented at hospital admission could serve as early indicators for readmission risk. Although no statistically significant differences in emergency room visit rates were found between groups in Türen & Enç [21], Suárez-González et al. [30] reported that incorporating specific nursing diagnoses into care plans for patients with respiratory diseases upon admission helps predict 30-day readmissions. This predictive capability could be particularly useful in managing chronic conditions like heart failure, where early identification of high-risk patients could allow for tailored interventions, ultimately reducing the burden on emergency and inpatient services.

This growing body of evidence underlines the importance of integrating frameworks such as Gordon’s FHP and nursing diagnoses into clinical practice, not only to improve patient outcomes directly but also to enhance the efficiency of care delivery through proactive management of nursing workload and targeted interventions.

Overall, using Gordon’s FHP has been proven to be useful in assessing patients’ experiences. The improvement in patient outcomes could be linked to critical thinking and decision-making. Using Gordon’s FHP could improve the accuracy of nursing diagnoses by making clearer diagnostic indicators (i.e., defining characteristics and related factors) for each diagnosis, using the Problem, Etiology, Signs and Symptoms (PES) format. This is consistent with a previous systematic review [2], which found that using SNTs was associated with greater effectiveness in critical thinking and clinical decision-making.

### 4.3. Integration of Nursing Assessment into Electronic Health Records

This review indicates a gradual increase in the integration of Gordon’s FHPs into EHRs, with more than half of the identified studies using electronic documentation, most conducted in the last decade. This trend suggests a shift from paper-based nursing assessment toward electronic systems that capture structured nursing data. These findings align with a previous review [8], which reported that although EHR use for SNTs was historically limited, adoption has increased over time as nursing documentation has become more standardized and digitally supported.

The growing use of EHRs in studies applying Gordon’s FHPs may enhance the systematic collection, aggregation, and analysis of nursing assessment data, thereby supporting the evaluation of nursing-sensitive outcomes and strengthening evidence-based nursing practice. Moreover, embedding structured nursing data in EHRs lays the foundation for Clinical Decision Support Systems (CDSS), which have been shown to improve diagnostic accuracy and clinical reasoning when grounded in SNTs and FHP-guided assessment [29]. A recent study on an AI-based CDSS for nursing diagnoses shows such models rely on structured, standardized data to support diagnostic reasoning, not replace nurses’ judgment. It highlights the importance of theory-based assessment frameworks for data quality and relevance [31].

### 4.4. Methodological Characteristics of Studies

The methodological profile of studies using Gordon’s FHPs shows substantial variability, with a predominant focus on associative analyses rather than causal or predictive inquiry. This methodological profile mirrors patterns previously described in research on SNTs, where studies initially relied on descriptive and associative analyses to characterize nursing care and assess feasibility, with a gradual shift toward more advanced multivariate and predictive methods as structured nursing data became more widely available in EHRs [8,27,32].

The limited number of randomized controlled trials highlights a persistent gap in high-level evidence to support causal inferences about the effects of FHP-guided nursing assessment. Although the consistent use of FHPs as independent variables underscores their applicability as structured assessment frameworks, the heterogeneity in analytical approaches and study designs limits comparability across studies. Future research would benefit from a greater use of experimental and longitudinal designs to strengthen causal evidence and better elucidate the contribution of FHP-based nursing assessment to patient and organizational outcomes.

### 4.5. Implications for Nursing Practice

Gordon’s FHPs provide a comprehensive framework for assessing patient health and guiding nursing care. This scoping review highlights the role of Gordon’s FHPs in enhancing the quality of nursing assessment, improving the accuracy of nursing diagnoses, and contributing to better patient and organizational outcomes. Our scoping review could provide information to clinical practitioners, researchers, policymakers, and, indirectly, nursing educators. Further studies could focus on unexplored outcomes and on educational outcomes not investigated in this review.

### 4.6. Strengths and Limitations

A major strength of this study is its contribution to addressing an important gap in the literature by providing a detailed, up-to-date synthesis of the association of Gordon’s FHPs on healthcare outcomes. Nevertheless, several limitations should be considered when interpreting the findings of this review. Firstly, we searched the literature across three databases, including English, Italian, and Portuguese-language articles. Despite the methodology’s accuracy, relevant studies may have been missed due to the selection of specific databases and languages. Secondly, in most studies, FHPs were not used in isolation but embedded within broader interventions (e.g., combined with SNTs or educational components), making it difficult to attribute observed outcomes solely to the FHP-based assessment. Thirdly, no quality assessment was conducted due to the study’s inherent methodology. Fourthly, there was heterogeneity in the outcomes investigated and in the different tools used. In addition, due to the limited literature, specifically on FHPs, we were unable to compare our findings with previous studies. To address this issue, the results were compared with research on standardized nursing language systems and studies utilizing NANDA-I. It is worth noting that none of the studies analyzed in this investigation showed a negative effect of using Gordon’s FHPs on outcomes. In addition, the small number of included studies (n = 7) limits the robustness and generalizability of the conclusions. The limited number of included studies reflects a genuine gap in outcome-based research on Gordon’s FHPs rather than restrictive inclusion criteria. Furthermore, one study included more than 51,000 participants, creating a marked imbalance in sample sizes across studies. This may disproportionately influence the overall synthesis, particularly regarding dependency-level findings. Therefore, interpretations should be made with caution, given the variability in study designs and sample sizes. The review may also be subject to publication bias, as studies reporting positive or statistically significant findings are more likely to be published and indexed. Finally, the geographical concentration of the included studies, predominantly from a small number of countries, may limit the broader international applicability of the findings.

## 5. Conclusions

This scoping review mapped the international literature on the use of Gordon’s FHPs and their associations with patient and organizational outcomes. Gordon’s FHPs were used across different clinical settings (e.g., hospitals and primary care) and multiple health contexts (e.g., heart failure and depression). Although it remains challenging to isolate the effects of Gordon’s FHPs on clinical outcomes due to heterogeneity in study designs and the predominance of associative analyses, the findings underscore the importance of a comprehensive and structured nursing assessment. Such assessments, as operationalized by Gordon’s FHPs, are foundational for generating accurate nursing diagnoses and informing evidence-based interventions that support both patient and organizational outcomes.

An important finding is the increasing integration of FHP-based assessments into EHRs, often in combination with SNTs (e.g., NANDA-I, NOC, NIC). This digital adoption enhances interoperability, facilitates structured, computable nursing data, and supports the development of CDSS, including AI-enabled approaches, to improve care planning, risk prediction, prognosis, and outcome monitoring. Strengthening the interoperability between FHP-based nursing assessments, EHR infrastructures, and AI-enabled CDSS can further enable advanced analytics, real-time feedback, and quality-of-care improvements across healthcare settings.

Despite these promising findings, the evidence base remains limited. There is a need for more high-quality, longitudinal, and predictive/prognostic studies to robustly evaluate the causal impact of FHPs on outcomes. Finally, future research should explore how interoperable EHR data and intelligent CDSS could optimize the use of FHPs in routine clinical practice, improve nursing diagnosis accuracy, and enhance patient-centered and data-driven care.

## Figures and Tables

**Table 1:** Population, Concept, and Context (PCC) framework.

PCC Component	Inclusion Criteria
**Population**	Nursing practice using Gordon’s FHPs
**Concept**	Patient and organizational outcomes
**Context**	Any nursing care setting

Abbreviations: FHP, functional health patterns.

**Table 2:** Main characteristics of included studies.

First Author, Year, Country, Design	Setting, Sample (IG: Intervention Group vs. CG: Control Group)	SNT	Health Record Type	Modality of Processing	Patient and Organizational Outcome (Measurements) & Follow-up Time (Length of the Study)	Key Results	**Main Type of Analysis (Test)**
Cazares Miranda, 2017, México, RCT [20]	Hospital (neurological department) Randomized sample of 171 adult outpatients with neurological diseases (IG: 100 Nurse counseling vs. CG: 71 Conventional inpatient care)	**X**	**X**	Educational intervention (Personalized Nurse Counseling) based on Gordon’s FHPs	**Pat:** Independence level (Barthel index), Treatment adherence (Morisky Green Scale), Timely physical rehabilitation, Identification of warning signs and symptoms, Recreational activity, Productive activities, With no urinary tract infection **Follow up:** T1: baseline; T2: 6 months; T3: 12 months (1 year)	**Pat:** The IG (vs. CG) had a significantly higher score of treatment adherence (effect size, 74%; *p* < 0.05) and independence (effect size, 23%; *p* < 0.05); engaged in recreational activities (OR = 6.0; 95% CI; 1.27–4.72; *p* = 0.007), productive activities (OR = 4.0; 95% CI; 2.19–8.9; *p* = 0.001), recognition of warning signs and symptoms (OR = 9.5; 95% CI; 4.63–21.5; *p* = 0.001), received timely rehabilitation (OR = 13.37; 95% CI; 4.56–86.82; *p* = 0.001), and had less urination problems (OR = 3.8; 95% CI; 1.89–7.8; *p* = 0.001)	Multivariate (adjusted logistic regression, ANCOVA) Cause-Effect
Türen, 2020, Turkey, RCT [21]	Hospital Randomized sample of 120 adult patients with heart failure (IG: 60 nursing care according to Gordon’s FHP model vs. CG: 60 standards nursing care)	**X**	**✓** Electronic	Booklet—Gordon’s FHP model	**Pat:** Quality of life (Minnesota Living with Heart Failure Questionnaire)/30-day mortality rate post-discharge (patient or relative + hospital medical records) **Org:** Emergency room visit; hospital readmission rates at 30th day. **Follow-up:** T1: baseline; T2: 30 days	**Pat:** The IG (vs. CG) had a significantly higher quality of life mean score at 30 days (62.3 ± 22.9 vs. 40.2 ± 23.5, *p* = 0.001). Kaplan–Meier survival curve analysis revealed a significant difference in the 30-day event-free survival rates between groups (log-rank *p* = 0.31), while the mortality rate was lower in the experimental group but without reaching a significant difference (*p* = 0.29). **Org:** Morbidity emergency room visits were higher in the control group but there was no statistically significant difference between the IG and the CG (*p* = 0.12). The IG (vs. CG) had a significant reduction in hospital readmission rates at 30th day (IG: 7; 11.7% vs. CG: 17; 28.3%; *p* = 0.02)	Multivariate (multivariate Cox regression) Cause-Effect
Temel, 2015, Turkey, CBA [22]	Hospital (psychiatric clinic) Convenience sample of 68 adult patients with depression (IG: 34 Gordon’s FHP model vs. CG 34 Routine program)	**✓** NANDA-I	**X**	Personal Care	**Pat:** Depression (Beck Depression Inventory), Hopelessness (Beck Hopelessness Scale), Self-efficacy (Depression Coping Self-Efficacy Scale) **Follow-up:** T1: baseline; T2: 3 months	**Pat:** The IG (vs. CG) had a significant decrease in depression symptoms and hopelessness and an increase in self-efficacy (*p* < 0.000)	Bivariate (*t*-test, chi-squared test, Mann–Whitney U, Wilcoxon signed-rank, Friedman test) Association
Cardenas-Valladolid, 2012, Spain, PCS [23]	Primary health care centers (n = 31) Convenience sample of 23,488 patients over 30 years of age with type 2 diabetes mellitus (two groups: Standardized nursing care plans (SNCP): 5168; usual nursing care (UNC): 18.320)	**✓** NANDA-I + NIC	✓ Electronic	Standardized nursing Care Plans	**Pat:** Glycemic (HbA1c), blood pressure (DBP), lipid (LDL-cholesterol), and weight (BMI) control **Follow up:** T1: baseline; T2: 12 months; T3: 18 months; T4: 24 months (2 years)	**Pat:** After adjusting for baseline parameter values, age, sex, type of treatment and physical inactivity, all parameters (except HbA1c) were lower, but a significant reduction was only observed with DBP results (but adjusted reduction in DBP of little clinical relevance). At T4, after adjusting for baseline parameters, the SNCP showed greater differences for control values of DBP, HbA1c, LDL-cholesterol and BMI but only reached statistical significance for HbA1c (*p* = 0.01). In the SNCP (vs. UNC), a greater proportion of patients with baseline HbA1c ≥ 7 decreased this value below 7% (16.9% vs. 15%; *p* < 0.01). The logistic regression model was adjusted (by type of treatment, age, and gender), and the SNCP demonstrated a favorable trend toward target control (OR = 1.11; 95% CI = 0.99–1.24; *p* = 0.06)	Multivariate (adjusted ANCOVA, logistic regression) Association
Lizcano-Álvarez, 2023, Spain, BA [24]	Primary health care centers (n = 40) Convenience sample of 132 adult patients with acute coronary syndrome (11 nursing consultation program based on Gordon’s FHPs)	**✓** NANDA-I + NOC	**✓** Electronic	Gordon’s FHPs (Actions linked to the corresponding NOC indicators), NANDA-I (00162—Readiness for Enhanced Health Management)	**Pat:** NOC Indicators scores: Self-management: Cardiac Disease, Compliance Behaviour: Prescribed Diet, Compliance Behavior: Prescribed Activity, Compliance Behaviour: Prescribed Medication **Follow-up:** T1: baseline; T2: 12 months (18 months)	**Pat:** Of the NOC indicators selected, all showed a statistically significant increase (*p* < 0.001) between before and after the intervention: Self-management: Cardiac Disease: (0.25 to 0.9 points); Compliance Behavior: Prescribed Diet (0.45 points); Compliance Behaviour: Prescribed Activity (0.33 points); Compliance Behaviour: Prescribed Medication (0.76 points)	Bivariate (Student’s *t*-test) Association
Brito-Brito, 2022 Spain, XS [25]	Primary health care center Convenience sample of 51,374 highly complex chronic adult patients	**✓** NNN	✓ Electronic	Gordon’s FHP model	**Pat:** A strong significant difference (*p* < 0.001) was observed between dependency levels and Physical activity/exercise, while a moderate significant difference (*p* < 0.001) between dependency levels and the following Patterns: Health perception/health management, Elimination, and Cognition/Perception **Follow-up:** NA	**Pat**: A strong significant difference (*p* < 0.001) was observed between dependency levels and Physical activity/exercise, while a moderate significant difference (*p* < 0.001) between dependency levels and the following Patterns: Health perception/health management, Elimination, and Cognition/Perception	Bivariate (chi-squared test) Association
O’Brien-Pallas, 1997, Canada, XS [26]	Hospital Convenience sample of 1435 pediatric and adult patients in medical, surgery, and specialty units	**✓** NANDA-I	**X**	Nursing diagnoses categorized using FHPs	**Org**: Nursing workload (Project Research in Nursing 80 instrument—direct care estimate) **Follow-up:** (113 days)	**Org**: A stepwise regression model (predictors: age, Canadian Case Mix Grouping, LOS, nursing diagnoses, FHP, Unit Factor scales, and nursing unit categories) explained 60% (F = 18.49, *p* < 0.001) of the variance in the nursing workload; this variance was explained by nursing diagnoses patterns (21%), age (9%), Case Mix Grouping (19%), LOS (5%), Unit Factor scales (3%), and nursing unit (3%). A positive linear relationship was noted between the number of nursing diagnoses selected within each Gordon’s FHP (except Coping-stress tolerance) and the average workload	Multivariate (ANOVA) Predictive

Note: Table 2 summarizes key characteristics of the included studies, including study design, setting, sample, SNTs used, health record type, nursing assessment approach, outcomes, key results, and analytical methods, to support interpretation across heterogeneous studies. **Abbreviations:** SNT, Standardized Nursing Terminologies. **Design**: RCT, Randomized controlled trial; CBA, Controlled before and after study; PCS, Prospective cohort study; BA, Before-and-after-comparison; XS, Cross-sectional study. **Modality of processing**: NANDA-I, NANDA-International; NIC, Nursing intervention classification; NOC, Nursing outcomes classification; NNN, harmonization of NANDA-I, NOC, and NIC; FHPs, Functional health patterns; SNCP, Standardized nursing care plans. **Outcomes**: BMI, Body mass index; DBP, Diastolic blood pressure; HbA1c, Glycated hemoglobin; LDL, Low-density lipoprotein; LOS, Length of Stay. **Main types of analysis:** ANCOVA, Analysis of Covariance; ANOVA, Analysis of Variance.

**Table 3:** Synthesis of patient and organizational outcomes ordered by the amount of evidence.

Patient’s Outcomes	Subcategories	First Author, Year
Clinical outcomes	Glycemic (HbA1c); blood pressure (DBP); lipid (LDL-cholesterol); weight (BMI) control; With no urinary tract infection	Cardenas-Valladolid, 2012 [23]; Cazares Miranda, 2017 [20]
Self-management/Compliance	Self-management: Cardiac Disease (NOC Indicators scores); Timely physical rehabilitation/Compliance Behaviour: Prescribed Diet, Compliance Behavior: Prescribed Activity, Compliance Behaviour: Prescribed Medication (NOC Indicators scores); Treatment adherence (Morisky Green Scale); Identification of warning signs and symptoms	Cazares Miranda, 2017 [20]; Lizcano-Álvarez, 2023 [24]
Dependency level	Barthel index; Classification of dependency level	Cazares Miranda, 2017 [20]; Brito-Brito, 2022 [25]
Mortality	30-day mortality rate post-discharge	Türen, 2020 [21]
Quality of life	Minnesota Living with Heart Failure Questionnaire	Türen, 2020 [21]
Psychological outcomes	Depression (Beck Depression Inventory), Hopelessness (Beck Hopelessness Scale), Self-efficacy (Depression Coping Self-Efficacy Scale)	Temel, 2015 [22]
Functional performance	Recreational activity; Productive activities	Cazares Miranda, 2017 [20]
**Organizational outcomes**	**Subcategories**	**First author, year**
Amount of nursing care indicators	Emergency room visit; Nursing workload (Project Research in Nursing 80 instrument—direct care estimate)	O’Brien-Pallas, 1997 [26]; Türen, 2020 [21].
Readmission	Hospital readmission rates on the 30th day	Türen, 2020 [21]

Abbreviations: BMI, Body mass index; DBP, Diastolic blood pressure; HbA1c, Glycated hemoglobin; LDL, Low-density lipoprotein.

## Data Availability

No new data were created or analyzed in this study. Data sharing is not applicable to this article.

## Supplementary Materials

The following supporting information can be downloaded at: https://www.mdpi.com/article/10.3390/healthcare14091144/s1, Supplementary file: Table S1: PRISMA checklist; Table S2: Key terms used in the search strategy.

## Abbreviations

The following abbreviations are used in this manuscript:

CDSS	Clinical decision support systems
EHRs	Electronic health records
FHPs	Functional health patterns
NANDA-I	NANDA-International
NIC	Nursing Interventions Classification
NOC	Nursing Outcomes Classification
SNTs	Standardized Nursing Terminologies
